# Enhancement of the Acid Resistance of Silty Clay Using Nano-Magnesium Oxide

**DOI:** 10.3390/ma16145035

**Published:** 2023-07-17

**Authors:** Areej Sadiq, Mohammed Y. Fattah, Mohammed F. Aswad

**Affiliations:** Civil Engineering Department, University of Technology, Baghdad 10066, Iraq; areejsadiq10@gmail.com (A.S.); mohammad_aswaad@yahoo.com (M.F.A.)

**Keywords:** acid resistance, hydrochloric acid, silty clay soil, contamination, nano-MgO, strength

## Abstract

Hydrochloric acid is prevalent in numerous industries; leakage of this acid may cause persistent problems in the soil. The study aims to prevent any adverse impact of acid on the strength characteristics of silty clay soil by modifying the soil’s acid resistance. In this study, unconfined compression tests are performed to investigate the strength of contaminated silty clay soil with concentrations of 4%, 8%, and 12% of HCl solution and the strength of treated soil with 0.4%, 0.5%, 0.6%, and 0.8% of nano-magnesium oxide. In addition, the strength of the soil enhanced with nano-MgO contaminated with different concentrations of hydrochloric acid was investigated to assess the effect of nano-MgO on modifying the acid resistance of clay soil. Moreover, the FE-SEM test was performed to analyze the microstructure of the soil under different circumstances. Based on the results, the strength of clay soil decreased due to contamination with the hydrochloric acid solutions; the reduction in strength was more noticeable when the acid solution became more acidic. Adding 0.6% of nano-magnesium oxide enhances the strength by about 114%. Findings show that adding 0.6% nano-MgO to the soil before exposing it to hydrochloric acid can enhance its acid resistance; the strength of the treated soil with nano-MgO was better at resisting the acid than the untreated soil.

## 1. Introduction

The rise of industrial growth worldwide has brought about a concerning increase in environmental contamination. The occurrence of chemical leakage into the soil and its associated issues have been a subject of significant interest among researchers. Many cases of construction failure due to leakages of inorganic acid into the ground have been documented in numerous industrial complexes across various world regions. Ref. [1] presented the initial documentation of foundation failures in three industrial buildings due to acid and alkali leakage; as a result, the dissolution of the foundation layer materials led to significant settlements. Sridharan et al. [2] stated that the phosphoric acid leakage into the subsoil caused soil heaving, significant cracking, and damage to the pavement, floors, and foundations of the fertilizer plant in Kerala state. In addition, refs. [3,4,5,6,7] have examined the impact of inorganic acid on various soil qualities. According to all of these researchers, the leakage of inorganic acids leads to soil contamination; this contamination can cause significant changes to the soil’s engineering properties, such as its design tolerance, compressibility, and strength, resulting in construction problems for facilities built on such soil.

Therefore, numerous scholars have studied the impact of inorganic acids on the geological characteristics of the soil. Sunil et al. [8] inspected the variation in the strength of laterite soils by immersing the soil in various pH solutions (5, 7, and 8). The pH of the solution was observed for around 90 days using 12 N hydrochloric acid and 15 M ammonia. When the pH of the solution was kept at 5, the results revealed a reduction in compressive strength. The reduction in compressive strength may be due to the iron content falling from 31.62% to 20.10% after the end of the immersion period; decreased iron content results in diminished cementation properties or aggregate structure disintegration.

Umesh et al. [9] found that adding different percentages of sulfuric acid (2.5%, 5%, 10%, and 15%) to black cotton soil, dispersive soil, and red loamy soil caused a decline in their unconfined compressive strength. This decline in strength can be attributed to the breaking of internal links and loss of cohesion within the soils, and the reduction becomes more significant as the acid concentration increases. In addition, the minerals in the clay content and the acidity of the pore fluid significantly affect the stress–strain behavior of soils contaminated with acidic solutions over the long term [10].

Bakhshipour et al. [11] investigated the impact of acid rain on the residual soil. The result stated that great fluidity of acid and low pH cause a reduction in soil strength and an increase in the coefficient of permeability of the soil. The artificial acid rain weakened the links among clay particles, and the dissolution of minerals caused substantial changes in the mineral structure.

Lei et al. [12] conducted lab experiments to investigate the impact of hydrochloric acid on the microscopic and geotechnical characteristics of the dredged fill. The findings indicated that the soil’s compressibility increased significantly while its undrained shear strength decreased. The investigation of the impact of acid on the microscopic properties indicated that the particle morphology changed from sheet structures to needle-shaped and flaky. Hydrogen ions caused significant damage to the cementation among soil particles through corrosion, causing the soil’s microstructural properties to deteriorate and increase large-diameter pores.

Liu et al. [13] assessed the influence of acid on the geotechnical characteristics of undisturbed samples of loess soil. The acid concentrations used were 1%, 4%, 8%, and 12%. They observed that the Atterberg limit, permeability, and unconfined compression strength of loess soil decreased with increasing hydrochloric acid fluid concentration. Upon examination of the engineering response in consideration of microstructure, it was observed that the predominant face-to-face contacts are present in loess treated with HCl acid. The result led to the opinion that structural characteristics and cementation salt are crucial in the behavior of loess soil subjected to hydrochloric acid. Also, Xu et al. [14] and Sun et al. [15] investigated the effect of acids on the geotechnical properties of the loess soil.

As contamination rates continue to rise, researchers have tried to find solutions to acid-contaminated soil problems. Ref. [4] suggested treating factory foundation soil contaminated with phosphoric acid; they recommended washing soils with water to mitigate the mineralization of acid-containing rocks and safeguard the foundation soils against acidic substances. A laboratory study was conducted on field specimens that had undergone sulfuric acid percolation and found that ground injection with sodium silicate was the best option to remediate the contaminated specimens, according to Al-Khailany et al. [16].

Chavali [17] conducted oedometer tests to assess the effectiveness of phosphogypsum in controlling the volumetric alterations induced by acidic conditions in the soil. They tested two types of soil: black cotton and kaolin clay. The results showed that treating phosphoric acid-contaminated soil with phosphogypsum reduced swelling and compressibility; this was due to the substitution of some clay with phosphogypsum, which prevented the formation of high-volume minerals such as sarcopside and merlinoite. However, the treatment was not effective in soils contaminated with sulfuric acid.

Tong et al. [18] investigated the effectiveness of using three materials: super absorbent polymer (SAP), lime, and crushed concrete, in treating soil contaminated with hydrochloric acid, where they examined the alterations in cohesion and internal friction using triaxial consolidated undrained tests (CU). The results indicated that the three types of curing materials could increase the cohesion of contaminated soil to a level higher than that of intact clay, thereby enhancing the strength of the clay to some degree.

Various materials are employed to decrease the disadvantages of soil, like fly ash [19], urban waste glass powder-based geopolymer [20], lime-silica fume [21], graphene oxide [22], magnesium oxide [23], and cement [24]. However, cement production emits around 7% of CO_2_ emissions worldwide, contributing to environmental issues like global warming, according to Yu et al. [25]. To achieve the requirement for environmentally friendly building construction, the key novelties for additives are reducing resource consumption, reducing energy use, reducing greenhouse gas emissions, and restraining pollution [26]. Therefore, looking for new treatment materials to reduce the consumption of natural resources and energy looks promising. With the recent rapid advancement of nanotechnology, the utilization of nanomaterials has attracted significant attention. Soil improvement based on nanomaterials is considered environmentally friendly and results in minor subsurface disturbance compared to conventional grouting techniques, according to Huang and Wang [27]. In addition, nano-remediation can eliminate the need to excavate and transport polluted soil, as the remediation process occurs in situ [28].

Metal oxide nanoparticles like MgO are employed to adsorb and decompose substantial quantities of hazardous substances [29,30]. These exceptional characteristics can be attributed to their remarkably high surface areas and elevated concentrations of reactive defect sites [31]. On the surface of the nanoparticles, destructive adsorption occurs, leading to the chemical decomposition of the adsorbate and the elimination of its toxic properties [32]. The inorganic nanoparticle MgO has gained significant interest owing to its unique features, such as its high reactivity, ceramic nature, wide band gap of 7.8 eV, thermodynamic stability, low dielectric constant, and various intrinsic defects [33,34].

The study conducted by Taha et al. [35] demonstrated that nano-MgO is significantly more effective than regular MgO at increasing the strength of soft soil. In addition, Gao et al. [36] stated that nano-magnesium oxide is a unique and highly functional material with distinct chemical and physical characteristics compared to conventional magnesium oxide. In other research by Supin et al. [37], changes in preparation temperature substantially affect the photocatalytic efficacy and the magnetic and optical properties of nano-MgO.

Recently, the utilization of nano-magnesium oxide as an additive to enhance the strength of clay soils has been increasingly documented in the literature [38,39,40,41]. Moreover, nano-MgO employment to improve the dynamic performance of soil was investigated [36,42].

However, the effectiveness of using nano-MgO for soil preparation to resist acid remains unknown. Therefore, in the present study, various percentages of nano-MgO were added to clay soil, and the soil was evaluated in a series of unconfined compression tests. A representation of the circumstances of soil exposure to different concentrations of hydrochloric acid was also carried out on the specimens of clay soil to investigate the strength behavior of soil contaminated with hydrochloric acid. In addition, the soil enhanced with nano-magnesium oxide was soaked in different concentrations of hydrochloric acid to assess the efficacy of nano-MgO in limiting the effects of acid on the strength characteristics of the clay soil.

## 2. Experimental Materials and Methods

### 2.1. Materials

Silty clay soil samples were collected at depths between 0.5 and 2 m beneath the ground’s surface from a building site in southern Baghdad in the middle of Iraq. Laboratory experiments were conducted on the soil to determine the physical characteristics of the soil. Table 1 summarizes the characteristics of the natural soil.

**Figure 1:**
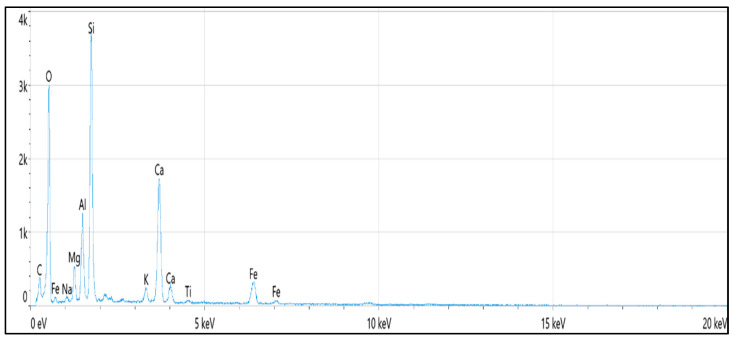
EDS analysis of the natural clay soil.

According to the Unified Soil Classification System (USCS), the soil is categorized as low-plasticity clay (CL). In addition, the energy-dispersive spectroscopy (EDS) test was utilized to identify the chemical elements in the studied soil. Table 2 and Figure 1 show the specific chemical elements of the soil.

This study focuses on a situation where hydrochloric acid has leaked. Based on prior studies conducted by Xu et al. [14] and Liu et al. [44], the concentrations (4%, 8%, and 12%) of the hydrochloric solution were chosen for the study. Specifically, by adding the concentrated acid to deionized water, the acid concentrates are prepared.

This investigation employed nano-magnesium oxide (nano-MgO) to enhance the strength properties of soil. The characteristics of nano-magnesium oxide were an average particle size range of 10 to 30 nm, a density of 3.58 g/cm^3^, a purity of 99.9%, and its appearance as a white powder. The selected percentage of nano-MgO used was 0.4%, 0.5%, 0.6%, and 0.8% of the total dry mass of the soil.

### 2.2. Specimens Preparation and Testing

In the present study, soil specimens were prepared using three major scenarios: soil contamination with different concentrations of HCl solution, natural soil treatment with varying percentages of nano-MgO, and exposing the soil treated with nano-MgO to different concentrations of HCl solution.

In order to prepare the specimens for the first scenario, the dry soils were first sieved through sieve #4 (4.75 mm) and then compacted in three layers with a maximum dry density (MDD) of 1.63 g/cm^3^ and an optimum water content (OMC) of 18.5% into PVC tubes that were 100 mm in diameter and 110 mm in height. In order to prevent soil loss during soaking, filter paper and wire cloth are wrapped around both ends of the soil specimens and fixed with a cable tie. Based on research by [10], seven days is sufficient for a chemical balance between the soil and the acidic solution. Therefore, the specimens were soaked in 4%, 8%, and 12% HCl acid solutions for ten days.

**Figure 2:**
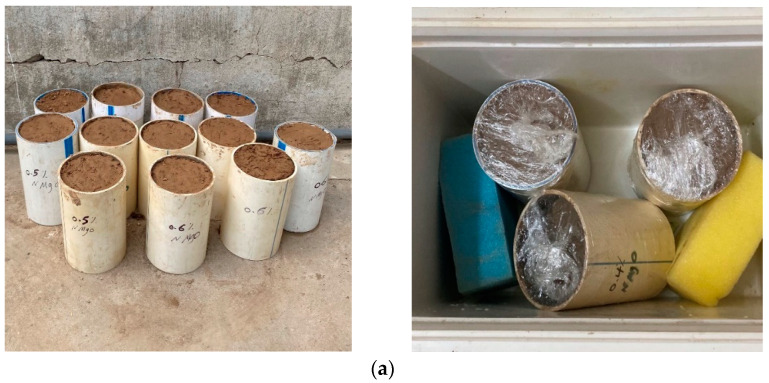

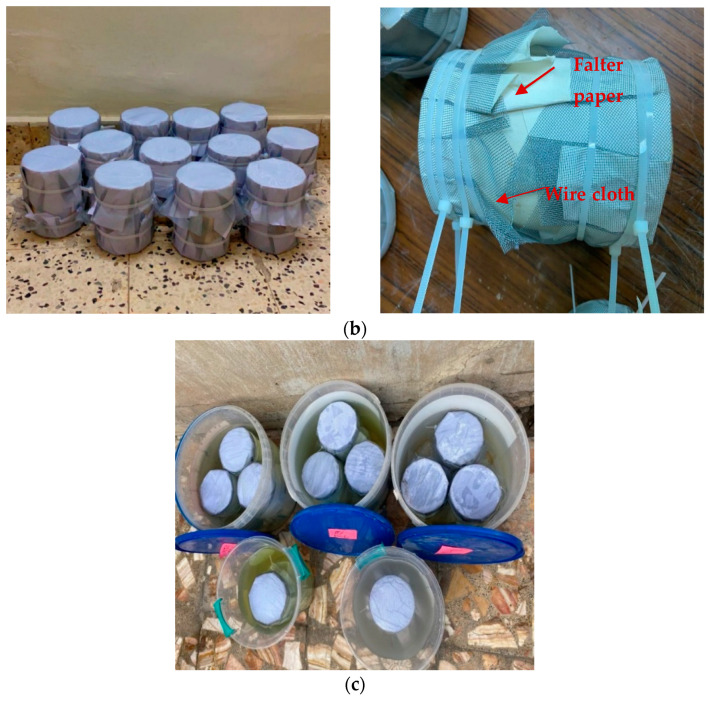
Preparation of Specimens. (**a**) Compaction of nano-treated soil specimens in PVC tubes and then curing in an airtight container; (**b**) all samples employed; and (**c**) soaking soil specimens with different concentrations of acid solution.

As for preparing the soil according to the second and third scenarios, the dry natural soil is crushed, sieved through sieve #4, and divided into layers. Then, predetermined amounts ranging from 0.4% to 0.8% of nano-magnesium oxide were sprayed on each layer; every layer was blended separately until a homogeneous mixture was obtained. The dry mix was agitated using a mechanical horizontal cylindrical mixer for ten minutes. The water amount determined based on the optimum moisture content was added step by step to the dry mixture until it reached the desired consistency. The mixture was compacted in three layers at the maximum dry density (MDD) in PVC plastic tubes; the specimens were then wrapped with plastic film and kept in an airtight container at laboratory temperature for seven days for curing. After curing, the soil specimens were separated into four groups. The first group was used as a reference for comparison purposes and was not contaminated with acid. At the same time, the other three groups were prepared to soak in different concentrations of HCl solution by wrapping both ends of the soil specimen in the same way as in the first scenario, then soaking the specimens in different concentrations of hydrochloric acid for ten days. Figure 2 illustrates the preparation of soil specimens. After the curing and soaking periods ended, hollow cylinders were instilled in the soil specimens (PVC tube). Then, the samples were extracted with a height of 76 mm and a diameter of 38 mm. According to ASTM D2166, unconfined compressive strength tests (UCS) were carried out under a vertical displacement rate of 0.5 mm per minute to explore the stress–strain relationship of the soil under three scenarios.

## 3. Test Results and Discussion

### 3.1. Unconfined Compressive Strength

#### 3.1.1. Impacts of Contamination Soil with Hydrochloric Acid

**Figure 3:**
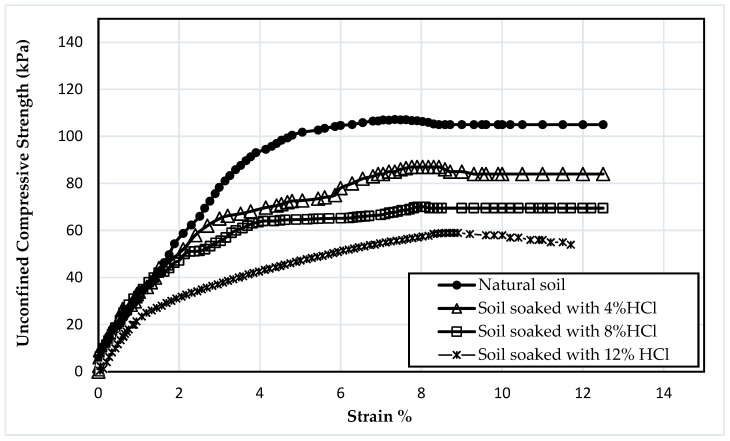
The stress–strain behavior of the natural soil under different concentrations of HCl solution.

Figure 3 demonstrates the stress–strain behavior of the natural soil subjected to varying concentrations of HCl acid solution (4%, 8%, and 12%). As revealed in the figure, the peak strength of natural soil yielded 107 kPa, while when the soil was soaked with 4%, 8%, and 12% HCl solutions, the peak strength decreased to 87.5 kPa, 70 kPa, and 58.2 kPa, respectively. The difference in results was associated with the concentrations of the acid solution, which indicates that as the concentration of the diluted acid rises, the reduction in shear strength is more notable. Table 2 shows that natural soil contains calcium (Ca), aluminum (Al), and iron (Fe), which create cementation between soil particles. However, soaking the soil in acidic solutions causes chemical reactions, as shown in Equations (1)–(3), which erode the oxides and break down the cementation between the soil particles; this is consistent with that mentioned by [45]: hydrochloric acid can dissolve iron oxides, carbonates, and the alumina octahedral layers of clay minerals. In addition, [11,12,46] mentioned that the soil’s fabric would be destroyed as the amounts of aluminum and iron decrease, reducing the soil’s shear strength.

CaCO_3_ + 2HCl → CaCl_2_ + H_2_O + CO_2_ (1)

Al_2_O_3_ + 6HCl → 2AlCl_3_ + 3H_2_O (2)

FeO + 2HCl → FeCl_2_ + H_2_O (3)

#### 3.1.2. Impacts of the Solitary Utilization of Nano-Magnesium Oxide

**Figure 4:**
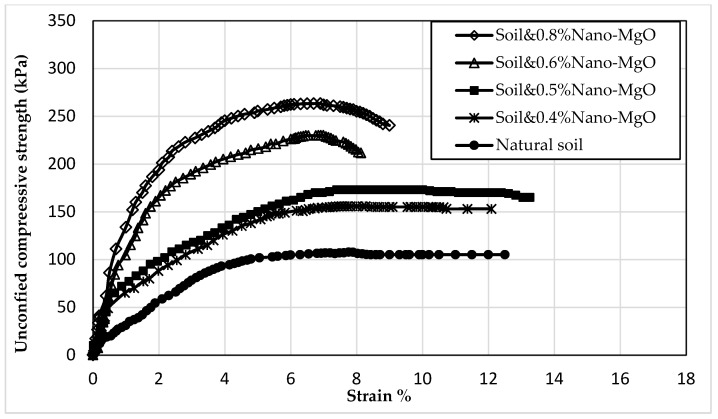
The stress–strain curve of the natural and nano-MgO-treated soil.

The set test involves one sample of natural soil and four samples of nano-treated soil; all the samples were tested after seven days of curing. The stress–strain relationship relating to unconfined compressive strength findings is depicted in Figure 4. It is evident from the figure that adding the nano-MgO to the soil, even at a low percentage, greatly improves the unconfined compressive strength (qu). The results indicated that the trend of increasing strength was linear. Adding 0.4, 0.5, 0.6, and 0.8% nano-MgO increased the qu value of the natural soil from 107 kPa to 155.7, 173, 230, and 261 kPa, respectively.

The soil composition typically includes solid particles, water, and air; the particles bound together to form the soil’s structure. The strength of the soil is primarily influenced by its density, void ratio, and water content. The enhancement in strength can be attributed to the cause that incorporating nano-MgO into the soil matrix reduces porosity owing to the nano-MgO filling of voids within the soil matrix and increases the connection between the particles, leading to enhanced and increased density. In addition, when nano-MgO is incorporated into the soil, chemical reactions between silicates (SiO_2_) in the clay and nano-MgO produce magnesium silicate hydrate gel, as shown in Equations (4) and (5). According to [41,47], this process makes soil particles more strongly bond together.

MgO+H_2_O → Mg^2+^+2(OH^−^) (4)

Mg^2+^+ 2(OH^−^) + SiO_2_ → Magnesium Silicate Hydrate (M-S-H) (5)

#### 3.1.3. Impacts of Soaking Nano-Treated Soil with HCl Acid Solution

**Figure 5:**
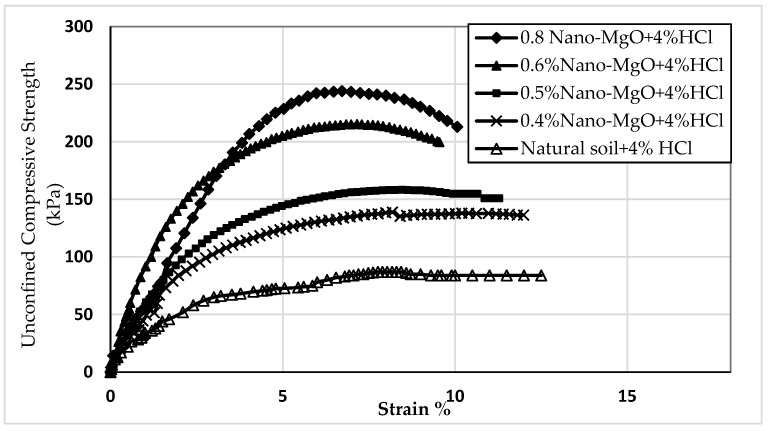
The stress–strain curves of nano-treated soil contaminated with 4% HCl acid.

**Figure 6:**
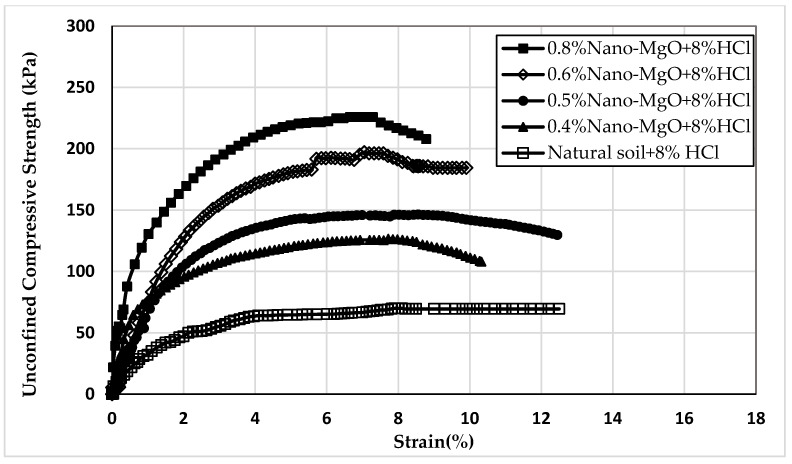
The stress–strain curves of nano-treated soil contaminated with 8% HCl acid.

**Figure 7:**
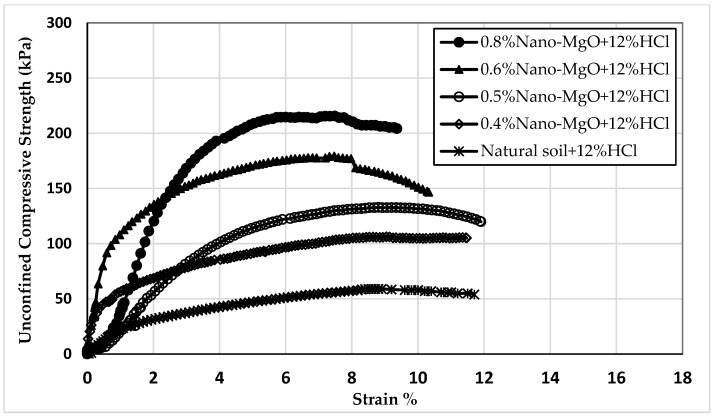
The stress–strain curves of nano-treated soil contaminated with 12% HCl acid.

**Figure 8:**
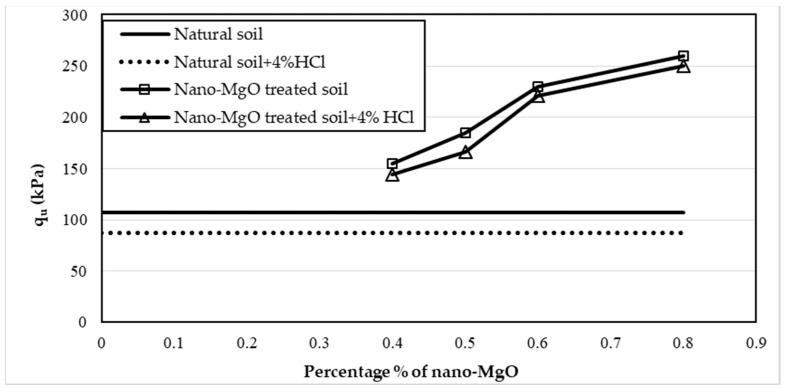
Variations of the unconfined compressive strength (qu) value of nano-treated soil contaminated with 4% of HCl solution.

**Figure 9:**
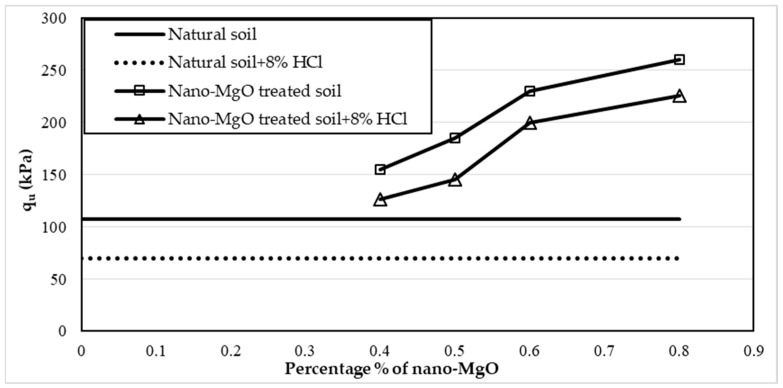
Variations of the unconfined compressive strength (qu) value of nano-treated soil contaminated with 8% of HCl solution.

**Figure 10:**
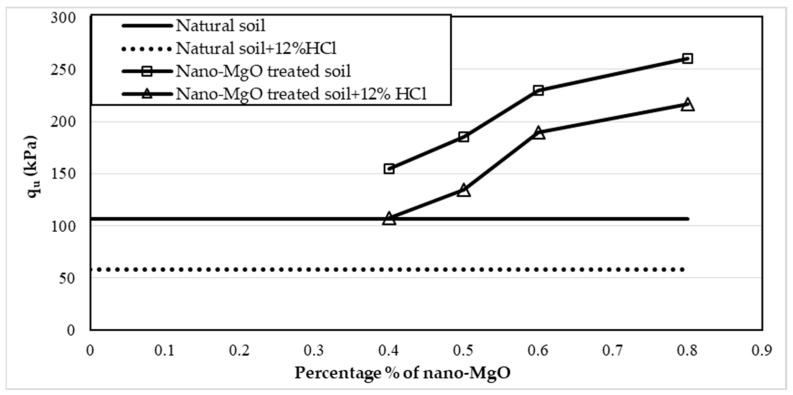
Variations of the unconfined compressive strength (qu) value of nano-treated soil contaminated with 12% of HCl solution.

Twelve samples prepared for the UCS test were evaluated to examine the influence of nano-MgO on modifying the acid resistance of clay soil. Figure 5, Figure 6 and Figure 7 show the stress–strain curves obtained from the UCS tests for the contamination of nano-treated soil with 4, 8, and 12% HCl solutions, respectively. Figure 8, Figure 9 and Figure 10 show the relationship between the unconfined compressive strength (qu) value and different percentages of nano-MgO in contaminated nano-treated soil samples with different concentrations (4%, 8%, and 12%) of HCl acid, respectively. Table 3 summarizes the results of the UCS test for the soil in different circumstances.

As displayed in Figure 5, Figure 6 and Figure 7, all 12 samples (nano-treated soil subjected to HCl solution) yielded a higher value of qu than the contaminated untreated soil. On the other hand, Figure 8 indicates that the value of qu of acid-contaminated nano-treated soil is very close to that of qu of nano-treated soil without acid. Moreover, when comparing them, the highest loss in the qu value was 7.5%, and the lower loss was 3.9%, obtained at 0.4% and 0.6% of nano-MgO, respectively, while when the natural soil was soaked in a concentration of 4% HCl acid, the decrease in qu value was 18.2%.

Figure 9 shows that the values of qu for the soil treated with (0.4, 0.5, 0.6, and 0.8%) nano-MgO and soaked with 8% HCl solution were 126.4, 145, 200, and 225.8 kPa. While the values of qu were 155.7, 173, 230, and 261 kPa for the nano-treated soil without acid, this demonstrates that the slight decline in the qu value of samples was in the range from 18.8 to 13.4%. While the untreated soil was soaked in the same acid concentration, the reduction in the qu value reached 34.4%.

Figure 10 displays that the qu values of samples with contents ranging from 0.4 to 0.8% of nano-MgO achieved higher values than qu for untreated soil when soaked with 12% HCl solution. On the other hand, the qu values for soil treated with nano-MgO at 0.5 to 0.8% and then subjected to 12% HCl solution were 133, 190, and 216.6 kPa. In contrast, the qu values for the nano-treated soil without acid were 173, 230, and 261 kPa; this means that the reduction in qu values was 23.7%, 17.1%, and 17% sequentially. When the sample with a percentage of 0.4% nano-MgO was soaked with acid, the qu of the soil equaled 107.5 kPa, which is close to the value of the natural soil and higher than the value of contaminated natural soil. In contrast, the qu value of the untreated soil exhibited a decline of more than 45.6% when soaked in the solution with the highest concentration of HCl (12%).

In general, the presence of nano-MgO provides greater acid resistance to the soil when compared to those without it, thereby mitigating the effects of environmental degradation. The behavior mentioned can be attributed to the following reasons: The nano-MgO particles used have a significantly smaller average diameter (10–30 nm) than the soil particles and significant ion exchangeability, owing to the higher chemical activity on the surface. It can be expected that nano-MgO reacts with acid instead of the elements that form soil cementation. In addition, the small dimensions and irregular surface of nano-MgO make it easy to form a strong bond among nano-magnesium oxide particles and easily agglomerate around the soil particles, which forms a film to protect the soil particles from the deleterious corrosion effects of acid.

According to the results obtained, adding an amount of 0.6% nano-MgO to the soil is appropriate as the optimal percentage for modifying the acid resistance of clayey soil. However, when comparing the sample treated with 0.6% of nano-MgO soaked in 4, 8, and 12% of HCl solution with the sample of soil treated with 0.6% of nano-MgO but not soaked, the percentage of loss in the value of the undrained shear strength was 3.9, 13, and 17.1%, respectively. While soaking the natural soil with the same concentrations, the loss in the value of qu was 18.2, 34.4, and 45.6%, respectively.

### 3.2. Mineralogical Analysis

**Figure 11:**
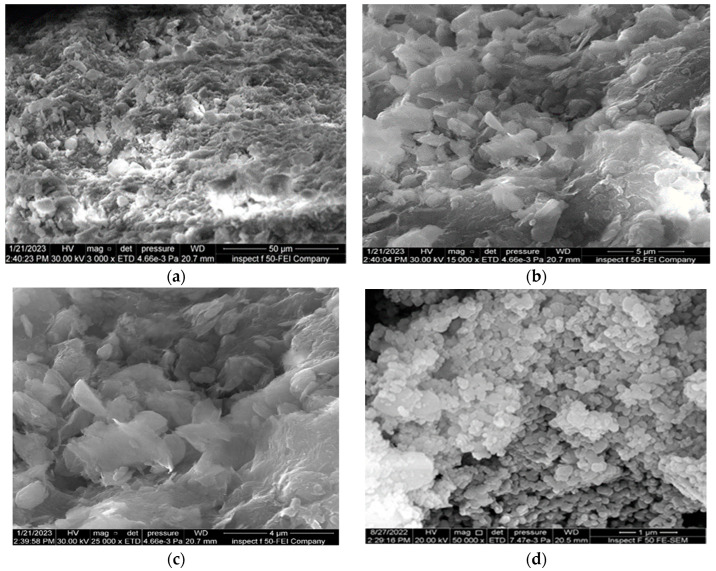
FE-SEM images of natural soil. (**a**) Zoom “3000” times; (**b**) zoom “15,000” times; (**c**) zoom “25,000” times; (**d**) nano-MgO zoom “500,000” times.

The field emission-scanning electron microscopy (FE-SEM) test was utilized to study the changes in the fabric of soil particles and microstructure characteristics under different conditions. The SEM findings revealed that the clay particles had a flaky distribution and sheet-like structure, with numerous micropores in the clay soil, as revealed in Figure 11.

**Figure 12:**
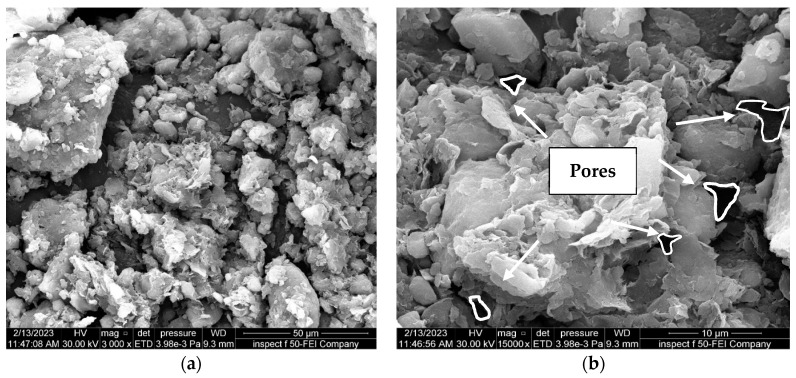
FE-SEM images of the natural soil contaminated with 8%HCl acid solution. (**a**) Zoom “3000” times; (**b**) zoom “15,000” times.

**Figure 13:**
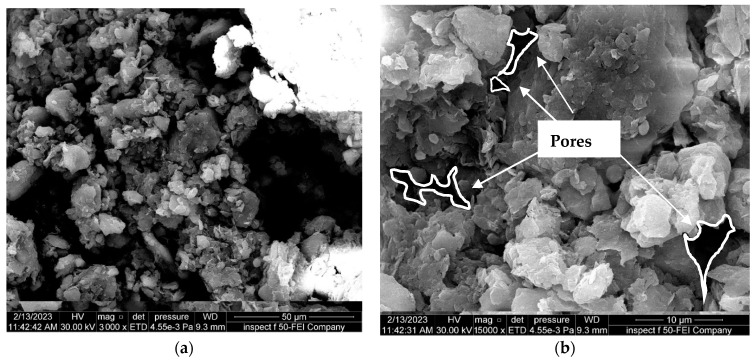
FE-SEM images of the natural soil contaminated with 12%HCl acid solution. (**a**) Zoom “3000” times; (**b**) zoom “15,000” times.

The effect of soaking soil in an 8% acid solution, as demonstrated in Figure 12, is the development of macropores between soil particles. Furthermore, as the HCl acid solution concentration increased to 12%, more pores formed and were distributed irregularly across the whole soil surface, and the dissolution fissures were visible, as depicted in Figure 13. These findings indicate that the chemical reaction mechanism between the acid and soil particles can lead to the dissolution of oxides and the loss of bond strength between soil particles.

**Figure 14:**
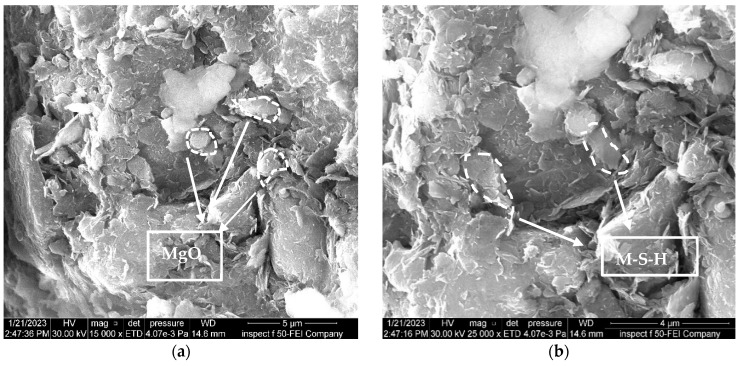
FE-SEM images of the natural soil treated with 0.6% nano-MgO. (**a**) Zoom “15,000” times; (**b**) zoom “25,000” times.

Figure 14 shows the effect of adding 0.6% of nano-MgO to the natural soil. When nano-MgO is introduced to the natural soil, it fills these pores and strengthens the connections between the various natural soil particles; furthermore, when nanoparticles are incorporated into the soil, a sequence of chemical reactions occurs to produce magnesium silicate hydrate gel. The effect of this process is that soil particles bond together more strongly, and the cementation among the soil particles is enhanced, thereby rendering the phenomenon of particle aggregation clearer.

**Figure 15:**
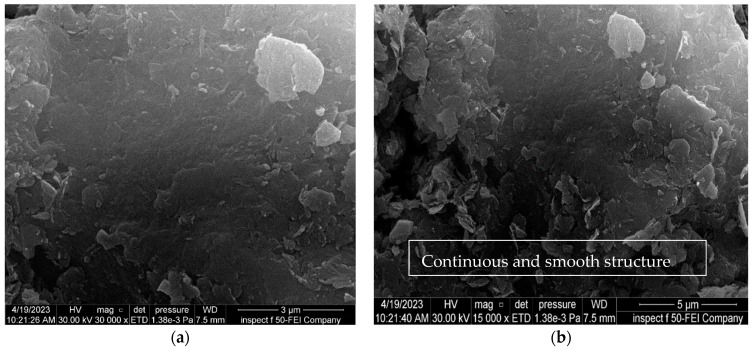
FE-SEM images of the soil treated with 0.6% nano-MgO contaminated with 4%HCl acid solution. (**a**) Zoom “3000” times; (**b**) zoom “15,000” times.

**Figure 16:**
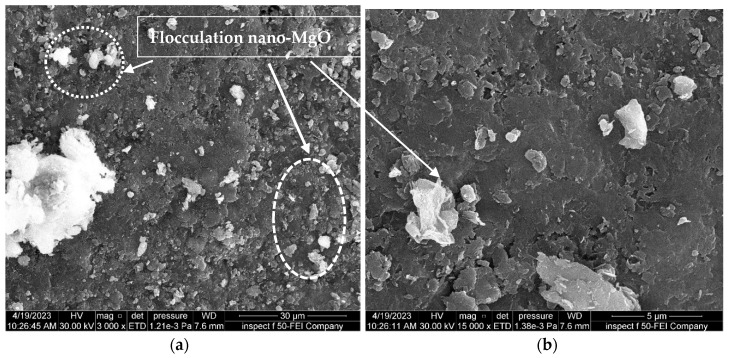
FE-SEM images of the soil treated with 0.6% nano-MgO contaminated with 8% HCl acid solution. (**a**) Zoom “3000” times; (**b**) zoom “15,000” times.

**Figure 17:**
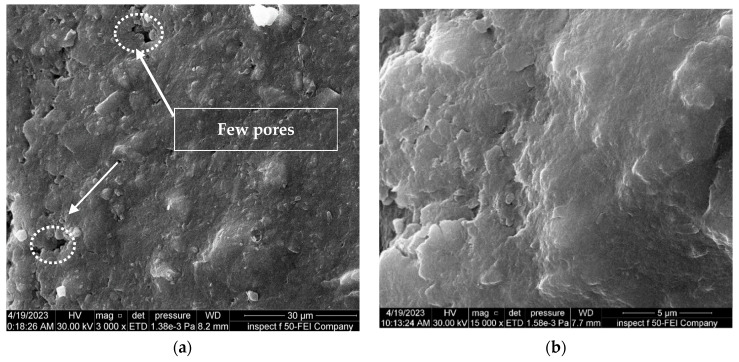
FE-SEM images of the soil treated with 0.6% nano-MgO contaminated with 12%HCl acid solution. (**a**) Zoom “3000” times; (**b**) zoom “15,000” times.

Figure 15, Figure 16 and Figure 17 show the effect of soaking the nano-treated soil with different hydrochloric acid concentrations (4, 8, and 12%), respectively. It can be observed that the shape of the soil surface exhibits continuous and smooth surfaces, the surface of the soil exhibits a limited number of micropores, and the degree of soil particle cohesion is satisfactory, as shown in Figure 15 and Figure 17. Furthermore, the flocculation of nano-magnesium oxide particles is distributed and scattered across the soil surface, which in turn fills voids within the soil, as revealed in Figure 16.

Furthermore, in comparison between the soil samples with and without nano-MgO, both of which were subjected to identical conditions and concentrations of contamination, the latter (Figure 12 and Figure 13) exhibited a significantly greater number of visible pores. Thus, these findings indicate that using nano-magnesium oxide is not conducive to enhancing the soil only but demonstrates significant efficacy in altering the acid resistance of clay soil because nano-magnesium oxide exhibits adsorption properties due to its significant surface area and ability to limit the effect of contaminants in soil.

## 4. Limitations of the Present Work

The present work cannot be considered a complete study of the treatment of clayey soil using nanomaterials. Other parameters that influence the behavior of clayey soil have not been taken into consideration in this work because of the limited time available and cost. Further studies are required to investigate the behavior of clay of different compositions and mineral compositions in addition to different percentages of nana MgO.

## 5. Conclusions

This investigation aimed to assess the effect of nano-MgO on the acid resistance of clay soil. The conclusions drawn from the results of the unconfined compressive strength tests and FE-SEM are as follows:

The peak strength of the natural soil generally decreased when it was soaked in a solution of hydrochloric acid; this is due to the many chemical reactions that occurred when the soil was soaked in an acidic solution, resulting in the dissolution of the oxides and the destruction of the cementation among the soil particles. The FE-SEM analysis indicated that the chemical reaction between soil particles and acid could form macropores between soil particles; as the HCl acid solution concentration increased, more voids developed, creating a weak soil structure.

Incorporating nano-MgO into natural soil can significantly enhance its unconfined compressive strength. The amount of nano-MgO added directly correlates to the strength increase in the soil.

Adding nano-MgO improved the clay soil’s ability to resist acidic conditions. The unconfined compressive strength (qu) of soil samples treated with 0.4% to 0.8% nano-MgO and soaked in 4%, 8%, and 12% HCl solutions was higher than that of untreated contaminated soil and natural soil. Because the nano-MgO particles used have a smaller average diameter (10–30 nm) than soil particles and significant ion exchangeability due to the higher chemical activity present on the surface, it is anticipated that nano-MgO will react with acid rather than the elements that form the cementation of soil. Using nano-MgO as a recommended additive is conducive to improving soil stabilization. It also modifies the acid resistance of clay soil.

## Figures and Tables

**Table 1:** Physical characteristics of the soil.

Physical Properties	Value	ASTM Specification Test [43]
Sand (%)	3	D422
Silt (%)	35	
Clay (%)	62	
Specific gravity, Gs	2.73	D854
Liquid limit (%)	43	D4318
Plastic limit (%)	19	
Plasticity index (%)	24	
Classification Soil	CL	D2487
Maximum dry density (g/cm^3^)	1.63	D698
Optimum moisture content, (%)	18.5	

**Table 2:** Chemical elements of the natural soil.

Element	Weight (%)
Oxygen, O	51.0
Magnesium, Mg	2.8
Aluminum, Al	5.8
Silicon, Si	16.4
Potassium, K	0.8
Carbon, C	8
Calcium, Ca	10.5
Titanium, Ti	0.3
Iron, Fe	3.4
Sodium, Na	0.6
Chlorine, Cl	0.2

**Table 3:** Results of unconfined compressive strength for the soil in different circumstances.

The Condition of the Soil	qu (kPa)	The Variation in qu (%)	The Comparison
Natural soil	107		
Soil + 4% HCl	87.5	−18.2	Compared with the natural soil
Soil + 8% HCl	70.1	−34.4	
Soil + 12% HCl	58.2	−45.6	
Soil + 0.4% nano-MgO	155.7	+45.5	Compared with the natural soil
Soil + 0.5% nano-MgO	173	+61.6	
Soil + 0.6% nano-MgO	230	+114.9	
Soil + 0.8% nano-MgO	261	+143.9	
Soil + 0.4% nano-MgO+4% HCl	144	−7.5	Compared with nano-MgO-treated soil
Soil + 0.5% nano-MgO + 4% HCl	164.4	−4.9	
Soil + 0.6% nano-MgO + 4% HCl	221	−3.9	
Soil + 0.8% nano-MgO + 4% HCl	250	−4.2	
Soil + 0.4% nano-MgO + 8% HCl	126.4	−18.8	Compared with nano-MgO-treated soil
Soil + 0.5% nano-MgO + 8% HCl	145	−16.1	
Soil + 0.6% nano-MgO + 8% HCl	200	−13	
Soil + 0.8% nano-MgO + 8% HCl	225.8	−13.4	
Soil + 0.4% nano-MgO + 12% HCl	107.5	−31.2	Compared with nano-MgO-treated soil
Soil + 0.5% nano-MgO + 12% HCl	133	−23.7	
Soil + 0.6% nano-MgO + 12% HCl	190	−17.3	
Soil + 0.8% nano-MgO + 12% HCl	216.6	−17	

## Data Availability

The data that have been used are confidential.
